# Supplementary material for: Causal Relationship between Aging and Anorexia Nervosa: A White-Matter-Microstructure-Mediated Mendelian Randomization Analysis
Source: Biomedicines. 2024 Aug 16;12(8):1874. doi: 10.3390/biomedicines12081874 (PMC11351342; doi:10.3390/biomedicines12081874)
Supplement: Supplementary file 1 [file biomedicines-12-01874-s001.zip › Supplementary Figure/Figure S4/Singe SNP plot of MR analysis for TL to ubm-b-1986.pdf]

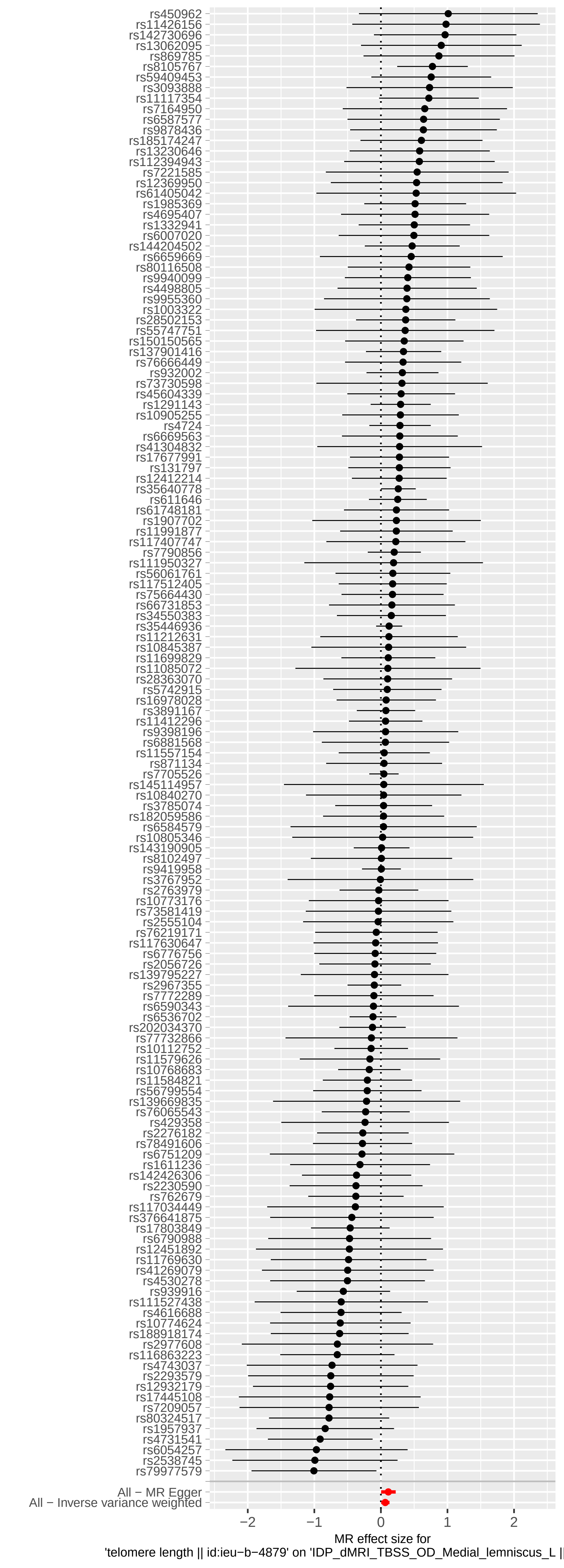
rs450962
rs11426156
rs142730696
rs13062095
rs869785
rs8105767
rs59409453
rs3093888
rs11117354
rs7164950
rs6587577
rs9878436
rs185174247
rs13230646
rs112394943
rs7221585
rs12369950
rs61405042
rs1985369
rs4695407
rs1332941
rs6007020
rs144204502
rs6659669
rs80116508
rs9940099
rs4498805
rs9955360
rs1003322
rs28502153
rs55747751
rs150150565
rs137901416
rs76666449
rs932002
rs73730598
rs45604339
rs1291143
rs10905255
rs4724
rs6669563
rs41304832
rs17677991
rs131797
rs12412214
rs35640778
rs611646
rs61748181
rs1907702
rs11991877
rs117407747
rs7790856
rs111950327
rs56061761
rs117512405
rs75664430
rs66731853
rs34550383
rs35446936
rs11212631
rs10845387
rs11699829
rs11085072
rs28363070
rs5742915
rs16978028
rs3891167
rs11412296
rs9398196
rs6881568
rs11557154
rs871134
rs7705526
rs145114957
rs10840270
rs3785074
rs182059586
rs6584579
rs10805346
rs143190905
rs8102497
rs9419958
rs3767952
rs2763979
rs10773176
rs73581419
rs2555104
rs76219171
rs117630647
rs6776756
rs2056726
rs139795227
rs2967355
rs7772289
rs6590343
rs6536702
rs202034370
rs77732866
rs10112752
rs11579626
rs10768683
rs11584821
rs56799554
rs139669835
rs76065543
rs429358
rs2276182
rs78491606
rs6751209
rs1611236
rs142426306
rs2230590
rs762679
rs117034449
rs376641875
rs17803849
rs6790988
rs12451892
rs11769630
rs41269079
rs4530278
rs939916
rs111527438
rs4616688
rs10774624
rs188918174
rs2977608
rs116863223
rs4743037
rs2293579
rs12932179
rs17445108
rs7209057
rs80324517
rs1957937
rs4731541
rs6054257
rs2538745
rs79977579
All − MR Egger
All − Inverse variance weighted
−2
−1
0
1
2
MR effect size for
'telomere length || id:ieu−b−4879' on 'IDP_dMRI_TBSS_OD_Medial_lemniscus_L ||
